# Treatment Options in Isolated Atherosclerotic Popliteal Artery Disease

**DOI:** 10.3390/jcdd12050170

**Published:** 2025-04-27

**Authors:** Stephanie Rassam, Raphaël Coscas

**Affiliations:** 1 Division of Vascular and Endovascular Surgery of the Department of Heart, Vascular and Endovascular Surgery, Paracelsus Medical University, 5020 Salzburg, Austria; 2 Department of Vascular Surgery, CHU Ambroise Paré, Assistance Publique Hôpitaux de Paris (AP-HP), 92104 Boulogne-Billancourt, France; 3 UMR 1018, Inserm-Paris11-CESP, Versailles Saint-Quentin-en-Yvelines University (Paris-Saclay University), Paul Brousse Hospital, 94807 Villejuif, France

**Keywords:** peripheral arterial disease, endovascular procedures, popliteal artery, vascular calcification, angiography, digital subtraction

## Abstract

Isolated popliteal artery (PA) lesions account for around 1% of lower limb revascularisations. Whatever treatment modality is chosen, the effects on the artery during knee flexion must be considered. The decision between a less invasive endovascular treatment (EVT) and traditional open interventions remains complex due to anatomical, biomechanical, and pathophysiological considerations and the varying aetiology of PA lesions. Available data remain limited, making it more challenging to decide on the most effective and durable treatment approach. Nowadays, when EVT is planned, several non-stenting techniques are available, making a “leave-nothing-behind strategy” possible after adequate vessel preparation. If stent implantation is required, self-expanding vasculomimetic stents are preferred due to their ability to provide flexibility and resist compression during motion. This narrative review discusses the available treatment options, challenges, and specific considerations for isolated PA disease, highlighting the need for large-scale, high-quality studies to provide more robust evidence on the optimal treatment approach.

## 1. Introduction

Isolated popliteal artery (PA) lesions account for around 1% of lower limb revascularisations in peripheral artery disease (PAD) lesions [1]. Occlusions and stenosis can lead to debilitating consequences for patients, particularly those with advanced disease. The management of isolated PA disease poses a challenge on different levels and has evolved over the past decade. Treatment strategies for popliteal artery (PA) disease range from open surgery to endovascular techniques. While open surgery such as thrombendarterectomy or interposition from a posterior approach or a bypass through a medial approach has been the standard treatment for years, endovascular treatment (EVT) has been partially accepted as a less invasive alternative, offering significant benefits in terms of recovery and patient outcomes [2,3,4]. The management of popliteal artery disease presents unique challenges, primarily due to the anatomical and pathophysiological complexity of the popliteal artery and its dynamic behaviour during knee flexion. The PA bends and moves as the knee flexes, resulting in biomechanical stresses that can impact the success of endovascular interventions, if not thoroughly planned. In addition, the PA has a narrowing diameter with the proximity of the bifurcation and tibial vessels and with a frequency of calcified lesions. Another aspect that needs to be considered is the aetiology of the presenting disease, which ranges from atherosclerotic to non-atherosclerotic lesions [1,5,6,7]. This is why knowledge, preoperative planning, and intraoperative obligations, such as dynamic angiography, are necessary to account for these challenges. While EVT has shown promising results for many patients with isolated PA repair, its durability has been criticised when compared to open surgical techniques. Open surgery in this area has been well established and offers a durable solution, particularly when autologous vein grafts are used. Open surgery comes with certain perioperative risks and prolonged recovery times and may not always be viable, especially in patients with limited conduit availability or other anaesthesiologic contraindications [8,9,10]. As a result, EVT continues to be an important alternative, particularly for those who seek or need a less invasive approach under the premise of technical feasibility. With a deeper understanding of the PA and the continuous advancements in endovascular techniques, EVT is likely to gain further importance in the management of PA disease. This narrative review presents the currently available treatment options and literature to strengthen the knowledge in this vascular field.

## 2. Methods

This is a narrative review of isolated popliteal artery repair. A literature search was performed using PubMed and Cochrane Library. The main search term was “popliteal artery”, and the following terms were searched in combination with this main search term and the Boolean operators AND or OR: “endarterectomy”, “endovascular”, “angioplasty”, “stenosis”, “occlusion”, “stent”, “balloon”, “peripheral artery disease” and via NOT, excluding “aneurysm”, “aneurysms”, “trauma”, “traumatic”, and “injury”. Of all the presented studies, without any time restrictions applied, one systematic review and meta-analysis, randomised controlled trials, cohort studies, and case series that analysed endovascular and open surgical repair via bypass or endarterectomy were included. This entailed comparative and single-arm studies. The provided references of each article were read through and added supplementarily, if feasible. English, German, and French studies were searched for inclusion. 

## 3. Overview

### 3.1. Anatomical Considerations

The popliteal artery (PA) begins after the passage of the femoral artery through the adductor hiatus. It passes through the popliteal fossa towards the lower border of the popliteus muscle, where it branches into its terminal vessels, the anterior and posterior tibial arteries. Originally, the PA was classified into three segments as a static anatomical concept: the P1 segment ranges from the intercondylar fossa to the proximal edge of the patella; the P2 segment, from the proximal part of the patella to the centre of the space of the knee joint; and the P3 segment, from the latter to the origin of the anterior tibial artery [11,12]. The extreme bending of the popliteal artery during flexion has been analysed in dynamic angiography. It has led to the definition of a “hinge point”, representing the main flexion area. Contrary to belief, it is not located at the joint line level but in the superior aspect of the PA, extending from the adductor ring to the superior border of the femoral condyle, thus entailing the P1 segment. This “hinge point” is accompanied by further “accessory flexion” that occurs proximal or distal to the described hinge point [13,14,15].

These observations raise the question of their knowledge and the importance of dynamic angiography for open surgical and endovascular repair. When the distal anastomosis in a lower-limb bypass is located juxta-articular or below the knee, the vein length has to anticipate the complete knee extension position to ensure a sufficient bypass length in motion. This can be verified with angiography. For endovascular repair, it is crucial to consider the landing zone of a potential stent. Deploying a stent at the level of the hinge point may result in a biomechanical constraint being transferred to the borders of the stent, thus making dynamic angiography necessary to adequately visualise potential kinks at the transition zones and the effect of movement on the stent. 

A pathophysiological aspect to consider is that the atherosclerotic affection of the PA presents differently from the iliac arteries, with circular fibres being more adherent to the adventitia, making disobliteration challenging [8,16].

### 3.2. Non-Atherosclerotic Disease

Non-atherosclerotic pathologies of the PA can cause claudication or critical limb-threatening ischaemia (CLTI). The most common causes are adventitial cystic disease and popliteal artery entrapment syndrome (PAES). Their diagnosis can be challenging, but age and a lack of or a few cardiovascular risk factors can be indicative of an atypical underlying disease. It can be diagnosed via ultrasound and necessitates further imaging with computer tomography angiography and functional magnetic resonance imaging [6,17]. In cases of a distal flow obstruction, angiography will be needed supplementarily. In cystic adventitial disease, diagnosis can be made based on the ultrasound performed when symptomatic; in further imaging, lateral displacement and stenosis, the scimitar sign, may be present [7]. In both pathologies, early diagnosis is paramount to shield the patient from serious consequences of distal flow.

Traditionally, resection of the affected area has been performed and reconstructed with a graft in cystic adventitia disease. Other treatment options include aspiration, resection, and evacuation of cysts with a risk of recurrence [6,7,17]. For PAES, treatment depends on the affection of the PA. Musculotendinous division without any vascular reconstruction will be sufficient in an undamaged vessel. Decompression via musculotendinous division and interposition of the PA is performed for compressions resulting in lesions of the PA. Femoropopliteal or femoro-crural bypasses are needed in cases of further disease extension beyond the PA [6,17].

Endovascular procedures have been singularly described before and may be used as temporary alternatives, although the underlying muscle entrapment or cystic adventitial impairment cannot be fully treated this way. 

### 3.3. Open Surgical Repair of the Popliteal Artery

Bypass surgery to surpass a popliteal lesion is a safe and effective option which requires an autologous vein graft for a superior outcome compared to using a prosthetic graft material. Depending on the surgical approach, the great saphenous (medial approach) or the small saphenous (posterior approach) vein can be easily harvested and used. Prosthetic, xenogeneic, or allografts may be used alternatively as a bypass material or as an interposition graft. The choice of conduit depends on its availability and experience. When a prosthetic graft is chosen, a ringed polytetrafluoroethylene graft is the conduit of choice [17,18]. A semi-closed endarterectomy via ring-stripper has been delineated in cases of adjacent superficial femoral artery disease [2,4,19,20] An open endarterectomy of short, isolated PA lesions is a feasible alternative. The procedure is generally performed through a posterior approach, which avoids muscle sections. The arteriotomy site can be reconstructed with different patch materials. The small saphenous vein can be used as an autologous option while enabling the preservation of the great saphenous vein [3]. An eversion endarterectomy and a primary closure have been described as another reconstruction technique in large diameters of the PA [2,19,20]. 

The postoperative course has been delineated with improvement subjectively and objectively and with patency rates of up to 89.4% at three years and 75.6% at five years [3,8,20].

Despite encouraging early and mid-term results, the literature on long-term data on this choice of procedure is scarce [2,8,19]. However, open surgery is not an option for all patients due to various factors such as conduit availability, surgical risk, and life expectancy.

### 3.4. The Percutaneous Treatment of the Popliteal Artery

Endovascular treatment (EVT) provides a viable alternative for many CLTI patients, offering significant improvements in limb salvage and amputation-free survival compared to untreated CLTI. Endovascular repair of the PA has been proven to be a safe and effective treatment option. Preoperative access and procedure planning are necessary for optimised results and are delineated in detail elsewhere [21,22]. The optimal treatment modality has not yet been delineated. Over the past few decades, the armamentarium of endovascular revascularisation techniques has expanded to include various non-stenting techniques for this critical anatomical zone. In suboptimal outcomes following percutaneous angioplasty, stenting is reserved as a bail-out option with direct improvement of the technical outcomes. This can be helpful in cases of flow-limiting dissections, significant residual stenosis, or recoil [9,23]. The literature on EVT for isolated PA disease remains limited, with most studies presenting short-term outcomes. This has made the interpretation of results challenging, particularly due to the frequent combination of popliteal with femoral artery disease under the broader heading of femoropopliteal disease, despite the distinct anatomical and biomechanical considerations that should be accounted for separately. One key aspect in this context is the potential need for stent implantation when a “leave-nothing-behind” approach is not feasible. In such cases, it is crucial to understand the deformation and biomechanical shear stress the stent will endure during knee motion to predict its long-term durability and performance [24].

Despite the challenges, endovascular treatment, including stent placement, offers promising results for patients with CLTI and complex PA lesions, especially for those not suitable for bypass surgery.

### 3.5. Vessel Preparation

#### 3.5.1. Plain Old Balloon Angioplasty

In endovascular therapy, plain old balloon angioplasty (POBA) is the most used technique, with inflation pressures ranging between 6 atmospheres (atm) in regular and over 30 atm in ultra-high-pressure balloon angioplasty. The latter is primarily used for fibrotic lesions and has traditionally been used for resistant stenosis. Traditionally, POBA has been used as a vessel preparation tool, without expecting full revascularisation in calcified lesions. Especially for below-the-knee (BTK) disease, it is preferred as a standardised treatment option because of its safety and effectiveness. Recent studies have shown that extended inflation results in superior outcomes compared to brief inflation, particularly in terms of reducing dissection and residual stenosis rate, which could potentially limit further treatment options and complicate further interventions. Trials comparing POBA to other EVT strategies in the PA have highlighted the potential benefits of long-term dilation in improving procedural outcomes [25,26]. Recently published studies have presented verifying findings when comparing EVT options for PA lesions. Böhme et al. reported a technical success rate of 83.3% (n = 50), with a patency rate of 71.7% (n = 43), 12 months post-procedurally in the POBA cohort, without a significant difference to patients treated with drug-coated balloon angioplasty (85.1% n = 40, *p* = 0.510) [27]. Bloch et al. analysed 689 endovascular procedures confined to the PA. Their results showed freedom from target lesion treatment failure of 76.8% (*p* = 0.293) in POBA compared to 72.6% for stenting, 81.2% for special balloon dilation, and 88.9% for atherectomy. These findings indicate that POBA may be less effective than other EVT modalities [9].

#### 3.5.2. Atherectomy

Atherectomy devices used in endovascular procedures aim to remove plaque and restore vascular flow, mirroring open surgery. These devices focus on the intimal layer of the artery to reduce stenosis and create a smoother vascular bed but cannot distinguish between soft tissue and calcification. Several types of atherectomy devices are available, each with specific characteristics suited for different clinical needs [28,29]. Despite their advantages, atherectomy procedures are often viewed with some caution due to potential periprocedural complications, such as vascular wall lesions and embolic events. Filter systems can be added to limit the risk of embolic events and are widely used in directional atherectomy; they are not required in orbital atherectomy. A detailed description of the devices and their preferred use are described elsewhere [21].

Semaan et al. compared atherectomy with PTA in the popliteal segment with comparable lesion qualities. After 12 months, freedom from reintervention was 75% in the atherectomy group and 76% in the PTA group. Another retrospective analysis comparing DCB alone with directional atherectomy (with distal protection device) with anti-restenotic therapy found a superior outcome for directional atherectomy with primary patency of 82% versus 65% (95% CI 1.09 to 6.37, *p* = 0.021) one year after the intervention. The TLR-free survival was comparable (94% versus 82%, *p* = 0.072). Different atherectomy devices were used [30]. Distal embolisation was more present in the study by Semaan (22% versus 0% PTA alone) than in the analysis presented by Stavroulakis (5% versus 3% for DCB alone, *p* = 0.99) [31].

A specific indication for debulking devices is the old embolic lesion, not diagnosed in the first 14 days of onset. In such cases, the debulking device may serve as an effective thrombectomy tool. Emboli are frequently stuck at the popliteal artery bifurcation due to the reduction in calibre. These lesions are highly challenging since they involve the bifurcation, where stenting is not desirable, and do not respond well to angioplasty due to their thrombotic nature. The Rotarex Rotational Excisional Atherectomy System (BD, Tempe, AZ, USA) has an interesting indication here, aiming to remove the old thrombus before using an anti-restenotic treatment [32,33]. Data focussing on the use of athero-thrombectomy in popliteal artery disease in cases of sub-acute and chronic occlusions are limited. Artzner et al. did not focus on the popliteal segments but demonstrated the possible use of athero-thrombectomy on the whole limb in cases of acute, subacute, and chronic occlusions in 297 cases, with their rates of primary patency being 69.6% (n = 111), 81.2% (n = 63), and 70.5% (n = 77), respectively [34].

#### 3.5.3. Intravascular Lithotripsy

Intravascular lithotripsy (IVL) offers a promising option for managing lesions with high calcification, with pulsatile sonic waves emitted through a traditional balloon catheter to fracture calcification in the intima and media. The resulting disruption of these plaques leads to lumen gain and improved vessel compliance, restoring elasticity. IVL is specifically designed for calcified lesions and can be used as a vessel preparation or a standalone procedure. Troisi et al. analysed popliteal lesions using the peripheral artery calcification scoring system (PACSS) and found that the majority of treated lesions had a score of 2 or higher, accounting for 72.3% of the 651 lesions treated [5]. A homogenous distribution in calcification severity was found in patients treated with stenting of the popliteal artery in a study by Scheinert et al. [11]. 

Regarding technical considerations, the catheter should be selected to be 10% oversized relative to the reference vessel diameter. The current peripheral IVL system, provided by Shockwave Medical (Santa Rosa, CA, USA), has low-profile catheter types S4 and E8 with a size range suitable for combined popliteal and BTK lesions. In the case of isolated PA disease, the M5+ catheter is generally the most suitable, with diameters up to 8 mm available. Successful vascular access and balloon inflation at low pressures of around 2 to 4 atmospheres are required. Importantly, no embolic filter is necessary for the procedure. IVL is associated with low periprocedural complication rates. Studies on IVL for the treatment of isolated PA disease remain limited. The DISRUPT PAD II and III trials include low proportions of popliteal lesions, with 26.7% of 60 and 18.3% of 153 patients, respectively, and will not be demonstrated. Stavroulakis et al. reported a higher proportion of PA lesions with 47.3% of the presented cohort of 55 patients. However, the primary patency of 81% reflects the outcomes of the entire cohort, making it difficult to draw proper conclusions on IVL in the PA. Nugteren et al. reported 30 limbs treated with IVL, of which over two-thirds were limited to the PA. Bailout stenting was required in 12.5% of the lesions, and the primary patency at 12 months was 68.8% [5,9,24,31].

#### 3.5.4. Cryoplasty

While Cryoplasty remains an underutilised treatment modality among vascular physicians nowadays, data on mid-term outcomes suggest it being a possible alternative treatment option. Through cryoplasty, the vessel-to-treat is frozen to –10 °C and has time to rewarm gradually. This changes the microstructure of the plaque and reduces elastic recoil by induced morphology changes, which result in short-term loss of vessel elasticity, enabling a more controlled distension. Another effect of cryoplasty is the apoptosis of vascular smooth muscle cells, which reduces the neointimal formation and synthesis of collagen. It has been postulated that cryoplasty may result in a more homogeneous response to the dilatation of a vessel. To date, high-level evidence for the procedures’ safety and efficacy is lacking [16,26]. A retrospective review of lower extremity EVT with cryoplasty by Schmieder in 2010 analysing data between 2004 and 2008 showed poor patency rates and clinical outcomes and highlighted the cost burden that might add to the challenge of a wide-spread use and thus larger datasets [35,36].

Pertaining to the femoropopliteal arteries, mid-term clinical outcomes have shown promising results, with primacy patency rates over 82% to 92%. In the PA, the results of a prospective, randomised single-centre trial comparing cryoplasty to conventional angioplasty conducted by Jahnke et al. showed a target lesion patency of 79.3% ± 7.5 at 9 months post-procedurally with a lower initial anatomic success rate in comparison to long-term dilation in conventional angioplasty. Complications were perceived in less than 5% [26]. 

For various reasons, cryoplasty is not very developed, and these devices are not available in routine clinical practice.

### 3.6. Anti-Restenotic Therapy

#### 3.6.1. Drug-Coated Balloon Angioplasty

This combined treatment modality of drug-coated balloons (DCBs) can directly treat soft atherosclerotic lesions. Its effectiveness may be limited in cases with a high atherosclerotic burden. Calcium will be a barrier to drug uptake. This is why in such cases, it is generally recommended to follow debulking or IVL strategies. This approach aims to prolong patency by delivering antiproliferative medication via the balloon’s drug coating to counteract the expected hyperplasia after EVT. The available options are paclitaxel- or more recently sirolimus-coated balloons. Preclinical studies in animal models have demonstrated that their synergistic effects have superior efficacy and safety profiles compared to single-drug coated balloon angioplasty [37]. These data warrant further investigation. 

The currently available literature presents the following findings regarding DCB angioplasty: A retrospective matched analysis by Böhme et al. compared DCB to POBA in PA lesions, demonstrating a higher target-lesion-revascularisation rate (TLR) of 93.6% in the DCB, compared to 81.7% in the POBA group (*p* = 0.060). The Endovascular Treatment of Atherosclerotic Popliteal Artery Lesions—Balloon Angioplasty Versus Primary Stenting (ETAP) trial reported a comparable TLR rate of 79.9% (23,27). A recent Korean prospective multi-centre registry published the 12-month outcomes of DCB treatment of popliteal lesions in 91 of 100 treated patients. Additional treatment modalities were required in 28% (n = 28) of patients, with atherectomy performed in 17% and provisional stenting in 11%. The primary patency rate at 12 months was 76%, and TLR-free survival was 87.2% [31,38,39].

#### 3.6.2. Stent Implantation

If balloon angioplasty alone does not achieve optimal results or if there is significant recoil or dissection, bailout stenting may be necessary. This is relevant in cases of inadequate flow following initial balloon angioplasty. The need for bailout stenting has been reported between 0.8% and almost 70% of the treated limbs [9,40]. Care must be taken when choosing the stent type in an area exposed to torsion, friction, and elongation. Table 1 provides a list of currently available stents as outlined in the Instructions for Use for the proximal or full popliteal segment [41].

**Figure 1:**
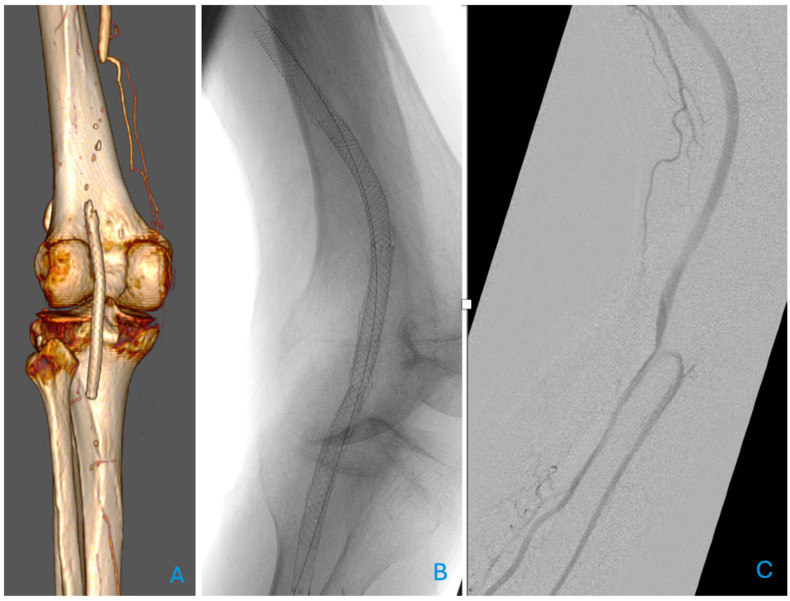
Relining of an occluded and fractured stent (not dedicated to the popliteal artery) (**A**) with a biomimetic self-expanding Supera stent (Abbott Vascular, Santa Clara, CA, USA) (**B**). The completion angiography with knee bending (**C**) is satisfactory.

The focus is on self-expanding stents which are particularly suited for the PA due to their high resistance, which is ideal for a segment in movement that will regularly be exposed to compression, i.e., when sitting. Vasculomimetic self-expanding stents are more advanced, with increased flexibility, low force exerted on the treated vessel, and improved fracture resistance. Examples are the Supera Stent (Abbott Vascular, Santa Clara, CA, USA) and the BioMimics 3D (Veryan Medical Ltd., Horsham, UK). The Supera stent is made from interwoven flexible nitinol wires, while the BioMimics 3D stent features a 3D helical geometric design. Vasculomimetic stents have been designed to reduce intimal hyperplasia. This is achieved by promoting a swirling flow, which increases wall shear stress and stabilises the anatomical challenges of this segment [11,23,42]. An example of a patient treated with a vasculomimetic stent is presented in Figure 1, where a relining of a fractured, unsuitable stent for the popliteal artery was necessary. 

The ETAP trial, a multi-centre randomised controlled trial, compared percutaneous transluminal angioplasty (PTA) with primary stenting in 246 patients. The stenting cohort received a self-expanding nitinol stent, a LifeStent Vascular Stent (Bard Peripheral Vascular; Tempe, AZ, USA). The study demonstrated superior direct technical success and a higher primary patency rate in the stent group when provisional stenting in the PTA group was considered as TLR. However, when provisional stenting was considered part of the PTA strategy, the 1-year patency rate was comparable between the groups. In 83.2% (n = 99) of the primary stenting group and 77.2% (n = 98) of the PTA group that were followed up at one year, the intention-to-treat analysis showed primary patency rates of 65.7% (n = 78) and 63% (n = 80), respectively, with secondary 1-year patency rates of 76.9% (n = 70) and 82.8% (n = 77) and 55.1% (n = 59), respectively. The intention-to-treat analysis revealed no significant difference in primary patency between the two groups [23]. The observed TLR rate of 20.1% was similar to the 18.3% reported by Böhme et al., who focused on DCB. Here, the occlusion rate was slightly lower [27]. The 2-year results published thereafter suggested a potential shift in favour of primary stenting due to higher patency rates, but mid-term and long-term results are absent to date [42]. In a study by Bloch et al., stent placement was found to be inferior to non-stenting EVT in terms of freedom from intervention failure (72.56% (n = 156/215) in the stent group versus 79.75% (n = 378/474) in the non-stenting group, *p* = 0.036) at one year. At this time, a restenosis rate of above 50% was observed in 12.56% (n = 27) of stent patients compared to 8.44% (n = 40) in the other intervention group. Subgroup analyses comparing bare metal stents to covered stent grafts did not show any superiority concerning freedom from intervention failure, reintervention, or residual stenosis, with a higher number of bare metal stents used (n = 171 versus n = 28) [9]. 

Scheinert et al. retrospectively analysed the outcome of 101 patients treated with the interwoven nitinol stent Supera. Primary and secondary patency rates were 87.7% and 96.5% at one year, respectively [11] Goltz et al. retrospectively studied 34 patients with CLTI treated with the same stent, revealing primary and secondary patency rates of 68.4% and 79.8% at one year, respectively. The TLR rate was 17.5%. 

The same stent was used in cases of PA occlusions in 86 patients, with a primary patency rate of 86.04% at 24 months. No stent fractures were observed on knee joint radiographs in the posteroanterior and lateral views [43].

An overview of the studies which solely focused on EVT for isolated PA repair due to PAD are presented with their study design, treatment modality, and outcomes in Table 2.

### 3.7. Endovascular Treatment Algorithm for Popliteal Artery Lesions

**Figure 2:**
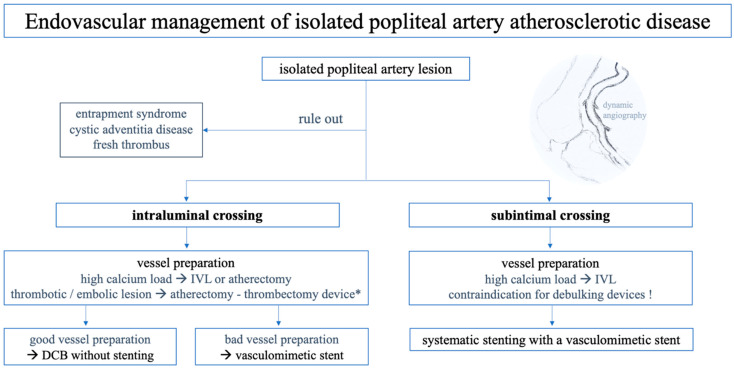
Endovascular repair of popliteal artery lesions in cases of peripheral artery disease, excluding acute occlusions. Dynamic angiography is always performed. (DCB… drug-coated balloon angioplasty; IVL … intravascular lithotripsy; * … Rotarex Rotational Excisional Atherectomy System (BD, Tempe, AZ, USA)).

Our treatment approach for popliteal artery repair is shown in Figure 2. The general risks associated with EVT, along with the specific challenges related to PA revascularisation, are outlined below to highlight further potential difficulties that may arise. Intraprocedural imaging with intravascular ultrasound may aid in understanding the presented pathology (e.g., lesion morphology, plaque composition, or stent apposition) or complications. Completion angiography with knee bending is always performed. 

### 3.8. Stent Fracture

Stent placement in the PA during endovascular revascularisation is controversial due to concerns over stent fractures, particularly given the high mechanical forces, bending, and foreshortening that occur with knee movement. These biomechanical stresses are known to contribute to stent failure and are exacerbated in cases of severe vessel calcification. The perceived axial compression in the PA has been indicated to be higher than in the adductor hiatus segment of the superficial femoral artery in all postures (*p* = 0.02). The average compression varies between 11% and 19% during femoropopliteal transition, and 13% and 25% in the PA [44,45]. Early studies, such as by Chang et al., reported a high stent fracture rate of 50% in PA when using older-generation nitinol stents. However, the advent of newer nitinol stents has significantly reduced fracture rates. In recent trials, stent fracture rates were reported between 3.4% and 7.1%, but no clear correlation between stent fracture and loss of primary patency has been established. Moreover, the relationship between stent fractures and restenosis or occlusion remains inconclusive [38,40,44,46] The most used stents have been analysed in a human cadaver model. The results demonstrated that all stents influenced femoropopliteal artery deformation during limb flexion in different ways. No stent was able to adapt to all deformation types without constricting or exacerbating the base of the arterial deformation [47].

Dynamic angiography to assess these forces, as recommended by Avisse et al. in 1995, can detect morphologic changes such as residual stenosis and recoil. Cui et al. reported a shift in treatment strategy in 26% of treated popliteal lesions following dynamic angiography. This change was attributed either to the initial morphology or morphologic changes after stent placement seen in dynamic angiography [48].

### 3.9. Embolic Events

The migration of plaque into the periphery or thromboembolic occlusions are complications associated with the EVT of PA lesions. In cases with a high risk of distal embolisation due to severe calcification or complex disease, the use of embolic protection devices, such as a distal filter, to prevent the release of plaque debris and subsequent microembolisation should be considered, also in consideration with the used treatment modality. The filtering strategy is challenging in P3 lesions close to the bifurcation since two arteries must be protected. Clots can be either aspirated via a catheter or with thrombectomy devices. When devices cannot aspirate a clot due to its size, alternative approaches like balloon maceration or stent placement may be considered to restore lumen and flow. However, stent placement below the tibiofibular trunk, surrounded by a thrombus, can be challenging regarding the remaining lumen, patency, and further migration. If the clot is well situated, a bailout strategy pushes it further, becoming more distal, into the peroneal artery, which can restore flow to the anterior or posterior tibial arteries.

Tissue plasminogen activator (tPA) installation as a bolus or infusion can be effective in certain cases, but the contraindications must be considered. In intra- or post-procedurally provoked acute limb ischaemia (ALI) with severe pain, tPA may not act quickly enough, necessitating immediate mechanical intervention. 

### 3.10. Distal Popliteal Lesions Involving the Bifurcation

In multi-level diseases involving both the PA and BTK arteries, pre-interventional planning is crucial to optimise technical success and, with that, the outcomes. A key aspect of managing these complex lesions is the simultaneous balloon dilatation in bifurcated lesions, as discussed in the femoral segment, to ensure a balanced result without compromising flow to either of the affected branches [21]. It may necessitate a larger femoral sheath and/or distal retrograde access to work with two wires and, at times, simultaneous balloons and stent inflations to sufficiently treat all the affected lesions or protect arteries not directly affected by the underlying disease.

## 4. Conclusions

EVT for PA atherosclerotic disease has demonstrated promising outcomes, offering a less invasive alternative to traditional open surgery, particularly in patients with high surgical risk. However, there remains a paucity of large-scale, high-quality studies comparing the mid- and long-term clinical outcomes, complications, and cost-effectiveness of endovascular versus open interventions for these lesions. Consequently, the decision-making process often hinges on factors other than evidence. To overcome these challenges and establish clearer treatment guidelines, well-designed randomised controlled studies should be planned, including diverse patient populations, standardised perioperative medical management and procedural techniques, patient-reported outcome measurements, and comprehensive follow-up protocols; the inclusion of anatomical, clinical, and economic endpoints will be essential to advance the understanding of the most suitable treatment modality for each PA lesion.

## Figures and Tables

**Table 1:** Stent options for endovascular treatment of the popliteal artery [41].

Company Name	Product Name	US FDA Indicated Use	Drug Delivery	Material Used	Introducer Size (F)	Maximal Guidewire Size (inch)	Stent Diameter (mm)	Stent Length (mm)	Delivery System Length (cm)
Biotronik, Inc.	Pulsar-18	SFA, proximal poplitea	–	nitinol	4	0.018	4–7	20–150	90, 135
Abbott	Supera Peripheral Stent System	SFA, proximal poplitea	–	nitinol	6	0.018	4.5–7.5 ”	20–150	120
Boston Scientific Corporation	Innova Vascular Self-Expanding Stent System	SFA, proximal poplitea	–	nitinol	6	0.035	5–8	20–200	75, 130
Veryan Medical	BioMimics 3D Vascular Stent System	SFA, proximal poplitea	–	nitinol	6	0.035	5, 6, 7	60–150	113
Boston Scientific Corporation	Eluvia Drug-Eluting Vascular Stent System	SFA, proximal poplitea	paclitaxel	nitinol	6	0.035	6, 7	40–150	130
Terumo Interventional Systems	R2P Misago RX Self-Expanding Peripheral Stent	SFA, proximal poplitea	–	nitinol	6	0.035	6, 7, 8	40–150	200
BD Interventional	LifeStent 5F Vascular Stent System	SFA, entire poplitea	–	nitinol	5	0.014–0.035	5, 6, 7	20–120, 150 *, 170 †	80, 135
BD Interventional	LifeStent, LifeStent XL Vascular Stent System	SFA, entire poplitea	–	nitinol	6	0.035	5, 6, 7	20–170	80, 130
BD Interventional	LifeStent Solo Vascular Stent System	SFA, entire poplitea	–	nitinol	6	0.035	6, 7	200	100, 135
Gore & Associates	Gore Viabahn Endoprosthesis With Propaten Bioactive Surface	SFA, proximal poplitea	–	nitinol/ePTFE, gold	6 to 10	0.018/0.014–0.035	5–11, 13	25–250	75 (0.035 system), 120

SFA: superficial femoral artery; US FDA: United States Food and Drug Administration; ” available in half and full sizes; * only available in 5–6 mm diameters; † only available in 5 mm diameter.

**Table 2:** Studies on endovascular treatment options for isolated popliteal artery lesions.

Author	Year	Study Design	Centres Involved	Lesions to Treat (n)	CLTI (%)	Vessel Preparation °	Anti-Restenotic Treatment °	Procedural Complication Rate (%)	Bailout Stenting (%)	Primary Patency (%)	Freedom from TLR (%)	Reintervention Rate (%)	Major Amputation Rate (%)
										*1 year*			
Scheinert [11]	2013	retrospective	single	101	22.8	PTA, DA +/−	stenting	11	−	87.7	NA	7	1
Rastan [23]	2013	prospective, randomised	multiple	127	20.7	PTA		2.4	25.2	65.7	55.9	4	0
				119		−	stenting	1.7	−	67.4	85.3	3	0
Stavroulakis [31]	2017	retrospective	single	36	16	PTA	DCB	5	16	65	94	22	0
				36	7	DA	DCB in all cases	5	5	82	82	14	0
Cui [40]	2017	retrospective	single	43	25.6	PTA		4.6	69	75.2	NA	NA	5.4
Rastan [28]	2018	prospective	multiple	162	30.4	DA	PTA +/−	15	3.6	75	78.8	9 *	0
Jahnke [26]	2020	prospective, randomised	single	40	27.5	−	cryoplasty	−	30	79.3 *at 9 months*			
				46	19.6	PTA		−	39	66.3 *at 9 months*			
Wu [39]	2021	retrospective, registry	single	54	NA	PTA, scoring +/−	DCB	13	14.8	72.6	NA	15	0
Donas [29]	2023	prospective	dual	62	37.1	PTA, DA	DCB +/−	6	4.8	NA	NA	NA	NA
Park [38]	2024	prospective, registry	multiple	100	severe CLTI excluded	AT +/−	DCB	4	11 provisional	76	87.2	NA	NA
Bloch [9]	2024	retrospective, registry	multiple	250	44.8	NA	PTA	NA	0.8	NA	76.8	14.4	4.8
				170	40	NA	DCB, cutting, IVL	NA	1.7	NA	81.2	11.8	3.5
				54	70.4	NA	AT	NA	20.4	NA	88.9	5.6	1.9
				215	39.5	PTA +/−	stenting	NA	7.4 thereof	NA	72.6	16.28	4.65
										*median follow-up up to 26 months*			
Troisi [5]	2022	retrospective	multiple ”	286	9.5	NA	PTA	3.8	−	77.4	75.2		4.8
				98	5.1	NA	DCB			69.3	77.9		2.5
				84	9.5	NA	Low−COF stent			65.3	68		2.7
				76	6.6	NA	High−COF stent			70.3	73		5.4
				17	11.8	NA	AT			89.5	89.5		2.6
				90	1.1	yes, DCB +/−	DA			61.5	76.9		0

AT: atherectomy; DA: directional atherectomy; NA: not available/assessed; TLR: target lesion revascularisation; COF: chronic outward force; DCB: drug-coated balloon; PA: popliteal artery; CLTI: critical limb threatening ischaemia; IVL: Intravascular lithotripsy; PTA: percutaneous transluminal angioplasty. ” comprising aforementioned data, * number could be higher, as numbers are bound to affected patients, not lesions, ° limited procedural data.

## Data Availability

Not applicable.
